# Minimal Change Disease and FSGS Are a Spectrum of a Single Disease within Immune-Mediated Nephrotic Syndrome

**DOI:** 10.34067/KID.0000000000000499

**Published:** 2024-07-09

**Authors:** John J. Sim, William E. Smoyer, Asher D. Schachter

**Affiliations:** 1 Division of Nephrology and Hypertension, Kaiser Permanente Los Angeles Medical Center, Los Angeles, California; 2 Department of Clinical Science, Kaiser Permanente Bernard J Tyson School of Medicine, Pasadena, California; 3 Department of Research and Evaluation, Kaiser Permanente Southern California, Pasadena, California; 4 Center for Clinical and Translational Research, The Research Institute at Nationwide Children's Hospital, The Ohio State University, Columbus, Ohio; 5 Visterra, Inc., Waltham, Massachusetts

**Keywords:** clinical nephrology, glomerular disease, glomerulopathy, nephrotic syndrome, podocyte, renal pathology

## Introduction

Minimal change disease (MCD) and FSGS are podocytopathies that are common causes of nephrotic syndrome (NS). Patients with MCD and FSGS demonstrate clinical heterogeneity that reflects the underlying pathogenic mechanism(s). For example, while FSGS is often used as a broadly encompassing histologic descriptive term, primary FSGS represents a small subset (<20%).^1^ Children with idiopathic NS are routinely presumed to have corticosteroid responsive NS, tending to correlate with MCD on biopsy. Taken together, MCD and primary FSGS are generally expected to respond to immunosuppression and considered immune-mediated. Thus, when considering podocytopathies that are induced by immune-mediated mechanisms (*e.g*., permeability factors), MCD and FSGS as disease entities start to converge.

MCD and primary FSGS as histologic descriptors reflect a common disease pathophysiology often referred to as immune-mediated FSGS (IM-FSGS) or presumed permeability factor-related FSGS. Including FSGS in both terms is an unfortunate remnant of the conventional assumption that histopathology alone can distinguish specific causes of podocytopathy. For the remainder of this perspective, IM-FSGS will be used interchangeably with primary and presumed permeability factor-related FSGS.

IM-FSGS can be defined as a composite of the following:

1. Extensive (≥80%) podocyte foot process effacement on electron microscopy.^1^
2. Nephrotic-range proteinuria or NS (defined as 24-hour urine protein >3.5 g/d and serum albumin <3.0 g/dl) and at least one episode of partial or complete remission with immunosuppression therapy (*e.g*., corticosteroids). This definition also includes the development of secondary resistance where proteinuria does not improve after an initial period of responsiveness/dependence to immunosuppression.
3. Absence of identifiable other causes (*e.g*., maladaptive/obesity-related, genetic mutation, drug-induced, viral infection, *etc.*).

FSGS is the common histologic end point for multiple glomerulopathies including conditions such as Alport syndrome and glomerular basement membrane diseases. Therefore, patients presenting with idiopathic NS but who have MCD on biopsy (*i.e*., no FSGS detected) should be presumed to have IM-FSGS. As such, the term IM-FSGS will be considered to include patients with immune-mediated minimal change disease henceforth.

### Clinical-Pathologic Considerations

**Figure 1:**
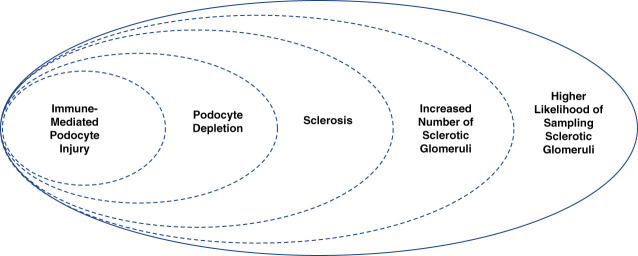
**Within immune-mediated NS, the histologic lesions of MCD and primary FSGS represent a common pathologic process and a single clinical disease entity.** MCD may be diagnosed earlier during the podocytopathy when sclerosis is less evident. With disease progression, podocyte loss and sclerosis of glomeruli become more prevalent, which also increases the likelihood of detecting FSGS in a biopsy sample. MCD, minimal change disease; NS, nephrotic syndrome.

MCD and primary FSGS are defined by the composite of extensive podocyte effacement and immune-responsive NS. In this model, earlier disease is more likely to demonstrate MCD on kidney biopsy, whereas later in disease progression, gradual sclerosis of glomeruli increases the likelihood of detecting FSGS in a biopsy sample (Figure 1). This contention is further supported by the observation that biopsies with FSGS have focal and segmental findings at the light microscopic level but demonstrate diffuse and global podocyte alterations at the electron microscopic level. Thus, a histologic diagnosis of MCD may reflect either of two scenarios: (*1*) sampling variance from a small sample of glomeruli (typically <20 glomeruli out of approximately 1 million present) that by chance failed to include glomeruli that were indeed present with FSGS or (*2*) an earlier stage of disease before more extensive involvement of the kidney and development of focal scarring within glomeruli.^2^ Variability in the degree of glomerular sclerosis can occur with different intensities of immunologic insult. For example, mild immunologic insults may initially alter podocyte signaling with alterations in the podocyte cytoskeleton, slit diaphragm compromise manifesting with selective albuminuria, and gradual sclerosis, whereas more intense immune-mediated insults may completely disrupt the cytoskeleton leading to podocyte destruction and glomerulosclerosis.

Histologic examination of transplanted kidney allografts with proteinuria recurrence has revealed an initial phase with MCD (normal appearing glomeruli on light microscopy, with extensive podocyte foot process effacement seen on electron microscopy). At later time points, FSGS has been observed in biopsies of those same kidneys .^3^ Serial biopsies of native kidney disease obtained early and later in the disease course demonstrated similar findings in both human and animal models.^4^

### Responsiveness to Corticosteroid Therapy Lends to Potential Mechanisms Linking MCD and FSGS

Among idiopathic NS, the specific histologic diagnosis (MCD versus FSGS) does not reliably predict critical clinical outcomes for patients, including steroid responsiveness, future disease course, or the risk of post-transplant recurrence. Although FSGS is considered more corticosteroid resistant and MCD more corticosteroid sensitive, the correlation is imperfect and conceptually simplistic. Patients with MCD have demonstrated frequently relapsing disease, corticosteroid dependence, and secondary corticosteroid resistance (after a period of corticosteroid responsiveness), whereas patients with FSGS have been reported to achieve complete remission with corticosteroid therapy.

In children, an initial course of corticosteroids will induce remission in approximately 90% of patients with MCD and 20%–60% with FSGS.^5^ However, approximately 60%–70% of patients with either MCD or FSGS relapse during corticosteroid tapering or after withdrawal. Thus, long-term maintenance corticosteroid therapy is often needed in combination with additional immunosuppressive drugs such as calcineurin inhibitors, alkylating agents (*e.g*., cyclophosphamide), rituximab, or mycophenolate mofetil to reduce the adverse effects of corticosteroid therapy. Rituximab therapy for either MCD or FSGS has similarly been reported to have 30%–50% relapse rates.^6^ Finally, children with MCD and initial corticosteroid sensitivity can develop secondary corticosteroid resistance, which is associated with higher risk of progressing to ESKD (up to 50% within 10 years).

IM-FSGS is also postulated to be due to a systemic circulating factor leading to podocyte dysfunction. This is evident in the high rate of recurrent disease in kidney allografts. Up to 30%–60% of patients can experience recurrence after transplantation. Among children with post-transplant recurrence of idiopathic NS, 93% had initial corticosteroid sensitivity compared with 30% who had corticosteroid resistance.^7^ One possible circulating factor is the discovery of autoantibodies directed at the critical podocyte slit diaphragm protein, nephrin. Transplant patients with recurrent FSGS have demonstrated high nephrin autoantibody levels which declined with remission.^8^ Moreover, nephrin autoantibodies have been described in 29%–44% of adults and children with NS due to MCD.^9,10^ These rates may be higher with the use of more sensitive assays and if patients were tested before immunosuppressive therapy.^10^ Targeted binding of antinephrin autoantibodies may disrupt nephrin structure (*e.g*., nephrin tyrosine phosphorylation) and/or function and lead to loss of slit diaphragm integrity, podocyte injury, and proteinuria.^10^ The presence of antinephrin autoantibodies were demonstrated to be higher if tested *before* treatment as immunosuppressive therapy may reverse the process, reducing or eliminating the circulating antibodies.^10^ In addition, screening children with immune-mediated NS found that 66% (among 341 patients) had one of seven different podocyte autoantibodies.^11^ Animal models have suggested that podocyte-specific dysfunction in signaling and/or structure can play a role as both an immunogenic stimulus and a pathogenic mechanism for podocyte injury. Such autoimmune processes can lead to a spectrum of injury including milder antibody-mediated processes or more inflammatory mechanisms with the possibility of complement activation. As reliable markers (*e.g*., podocyte-specific antibodies) are established to clearly identify and affirm IM-FSGS disease and mechanism, we hope such improved understanding of molecular pathophysiology will pave the way for acceptance of more distinct terminology.

## Conclusions and Recommendation

Several lines of preclinical, translational, and clinical evidence collectively support the hypothesis that immune-mediated NS can manifest histologically as MCD and/or FSGS, likely reflecting different stages of disease. Indeed, we believe that continued adherence to this histologic distinction will only hinder efforts to develop safer and more effective therapies for this devastating disease, affecting patient populations who could be most likely to benefit from more targeted immunomodulating and/or nonimmunomodulating therapies. We propose that classifying this disease based on its apparent molecular pathogenesis rather than its histology will provide a better foundation for both development of more targeted treatments and more precise management of patients with NS. Therefore, we recommend, using the term immune-mediated NS to encompass MCD, FSGS, immune-mediated minimal change disease, and IM-FSGS.
